# A hidden Markov model approach for determining expression from genomic tiling micro arrays

**DOI:** 10.1186/1471-2105-7-239

**Published:** 2006-05-03

**Authors:** Kasper Munch, Paul P Gardner, Peter Arctander, Anders Krogh

**Affiliations:** 1 Bioinformatics Centre, Institute of Molecular Biology and Physiology, University of Copenhagen, Universitetsparken 15, 2100 Copenhagen, Denmark; 2 Molecular Evolution Group, Institute of Molecular Biology and Physiology, University of Copenhagen, Universitetsparken 15, 2100 Copenhagen, Denmark

## Abstract

**Background:**

Genomic tiling micro arrays have great potential for identifying previously undiscovered coding as well as non-coding transcription. To-date, however, analyses of these data have been performed in an *ad hoc *fashion.

**Results:**

We present a probabilistic procedure, ExpressHMM, that adaptively models tiling data prior to predicting expression on genomic sequence. A hidden Markov model (HMM) is used to model the distributions of tiling array probe scores in expressed and non-expressed regions. The HMM is trained on sets of probes mapped to regions of annotated expression and non-expression. Subsequently, prediction of transcribed fragments is made on tiled genomic sequence. The prediction is accompanied by an expression probability curve for visual inspection of the supporting evidence. We test ExpressHMM on data from the Cheng *et al. *(2005) tiling array experiments on ten Human chromosomes [1]. Results can be downloaded and viewed from our web site [2].

**Conclusion:**

The value of adaptive modelling of fluorescence scores prior to categorisation into expressed and non-expressed probes is demonstrated. Our results indicate that our adaptive approach is superior to the previous analysis in terms of nucleotide sensitivity and transfrag specificity.

## Background

Tiling micro arrays query genomic sequence in a manner not biased towards coding transcripts and has proven a valuable resource in the exploration of genomic expression. To date, the approach has been applied to Human, Arabidopsis, and Rice [3-11] and has been reviewed recently [12,13]. The challenge of tiling micro array analysis is to categorise genomically consecutive probes as either expressed or non-expressed. The Affymetrix tiling array analyses [1,3,4] invoke extensive experience from gene arrays. Here, chip noise between replicates is dealt with using a quantile-normalisation procedure [14]. Background and probe sequence-specific signal is normalised using the MAS 5.0 method [15]. In order to convert expression values into transcribed fragments (transfrags) Affymetrix uses a smoothing window approach, whereby expression values within a genomic neighbourhood are averaged. A region is labelled as expressed if all probes exceed a threshold value. The threshold is determined from expressed RNA spiked into the micro array experiment. Neighbouring regions closer than a distance threshold are then joined, forming transfrag predictions. We will use the collective acronym ANTM for for Affymetrix's normalisation and transfrag methods. For a review of existing algorithms for tiling micro array analysis see Royce *et al. *[16].

Previous related work includes analysis of ChlP-chip experiments using Affymetrix tiling arrays to predict transcription factor binding sites [17]. These ChlP-enriched regions on the tiling arrays are predicted using log-odds scores for each probe generated by a two-state hidden Markov model. Tiling array intensities have been used together with a rudimentary HMM gene finder to establish likely exon-intron transcripts in windows of significant expression intensity [18]. This work, however, does not treat the probe intensities as observables of the HMM but as separate evidence supporting prediction of expression.

The approach to tiling array analysis presented here employs a hidden Markov model (HMM), trained directly on the correspondence between tiling array fluorescence scores and annotation of expression. The model is then used to categorise consecutive probes as expressed or non-expressed. Training the model using annotation of expression presents a problem since, in many cases, annotation does not represent actual expression. This may be due to a number of factors, such as non-expressed genes, alternative splicing, un-annotated exons, antisense transcription, and non-coding RNAs. This type of incomplete information presents a problem to machine learning approaches as missing information compromises the training of the model unless properly addressed. We describe a two-step modelling and training approach that allows the training algorithm to down-weight dubious annotation in the context of the relevant cell line. The adaptive nature of our method avoids the *ad hoc *thresholds characteristic of previous work. As a case study we analyse Human tiling array data of a single cell-line from Cheng *et al. *[1].

## Methods

### Data

The data we use is derived from the tiling array experiments on Human chromosomes 6, 7, 13, 14, 19, 20, 21, 22, X, and Y [1]. The tiling arrays use 25-mer probes querying genomic positions at five nucleotide intervals on the genomic sequence. Only non-repetitive sequence is tiled resulting in a coverage of approximately 30%. From the Cheng *et al. *raw array data we analyse the subset querying poly-adenylated RNA extracted from Human neuroblastoma cells (SK-N-AS cell line).

### Array and probe normalisation

The Affymetrix MAS 5.0 method addresses probe and array normalisation by, in addition to perfect match (PM) probes, also designing mismatch (MM) probes, where the complementary base at the 13th position of the 25 mer probe is used instead of the actual base. The mismatch probe is expected to capture much of the probe sequence and array-specific contributions to the signal as well as problematic probe eccentricities such as secondary-structure and cross-hybridisation effects while capturing little real signal. The difference in fluorescence score for the PM probe and an Idealised-MM probe score is then used to estimate expression signal from the queried genomic position.

We tested a number of strategies for array and probe normalisation. These included *PM *- *MM *with and without quantile normalisation and a novel approach inspired by Naef and Magnasco [19] that shares similarities with GC-RMA [20,21].

For the normalisation inspired by Naef and Magnasco sequence and array specific signal is modelled in a way that allows this to be subtracted from the raw signal. For each chip a large sample of probe scores are selected and used to fit position-specific estimates of nucleotide contribution to the signal. Three free parameters for each position times the 25 parameters for each probe sequence position gives rise to 75 parameters. These are modelled by the following linear system:

PM.estimate=∑i=175aixi,
 MathType@MTEF@5@5@+=feaafiart1ev1aaatCvAUfKttLearuWrP9MDH5MBPbIqV92AaeXatLxBI9gBaebbnrfifHhDYfgasaacH8akY=wiFfYdH8Gipec8Eeeu0xXdbba9frFj0=OqFfea0dXdd9vqai=hGuQ8kuc9pgc9s8qqaq=dirpe0xb9q8qiLsFr0=vr0=vr0dc8meaabaqaciaacaGaaeqabaqabeGadaaakeaacqWGqbaucqWGnbqtcqGGUaGlcqWGLbqzcqWGZbWCcqWG0baDcqWGPbqAcqWGTbqBcqWGHbqycqWG0baDcqWGLbqzcqGH9aqpdaaeWbqaaiabdggaHnaaBaaaleaacqWGPbqAaeqaaOGaemiEaG3aaSbaaSqaaiabdMgaPbqabaaabaGaemyAaKMaeyypa0JaeGymaedabaGaeG4naCJaeGynaudaniabggHiLdGccqGGSaalaaa@4A24@

where each nucleotide type (*A, C, G *or *T*) in position *k *in a 25-mer probe is modelled by a triple in the indicator function *a*_*i *_(*a*_3*k*-2_, *a*_3*k*-1_, *a*_3*k*_). I.e. an *A *at position *k *has corresponding coefficients (*a*_3*k*-2_, *a*_3*k*-1_, *a*_3*k*_) = (1, 0, 0), a *C *has corresponding coefficients (0, 1, 0), a *G *has corresponding coefficients (0, 0, 1), and a *T *has corresponding coefficients (-1, -1, -1). A least-squares solution to this system of equations for *x*_*i *_provides estimates of the expected contribution to the expression for any nucleotide at position *k*. That is, the estimated contribution to the expression level of an *A *at position *k *is given by *x*_3*k*-2_, a *C *at position *k *is given by *x*_3*k*-1_, a *G *at position *k *is given by *x*_3*k*_, and a *T *at position *k *is given by -*x*_3*k*-2 _- *x*_3*k*-1 _- *x*_3*k*_. To obtain a representative expression level over different replicates the mean log-likelihoods are computed:

LL:=13∑i=13log⁡2PMiestimate.PMi.
 MathType@MTEF@5@5@+=feaafiart1ev1aaatCvAUfKttLearuWrP9MDH5MBPbIqV92AaeXatLxBI9gBaebbnrfifHhDYfgasaacH8akY=wiFfYdH8Gipec8Eeeu0xXdbba9frFj0=OqFfea0dXdd9vqai=hGuQ8kuc9pgc9s8qqaq=dirpe0xb9q8qiLsFr0=vr0=vr0dc8meaabaqaciaacaGaaeqabaqabeGadaaakeaacqWGmbatcqWGmbatcqGG6aGocqGH9aqpdaWcaaqaaiabigdaXaqaaiabiodaZaaadaaeWbqaaiGbcYgaSjabc+gaVjabcEgaNnaaBaaaleaacqaIYaGmaeqaaaqaaiabdMgaPjabg2da9iabigdaXaqaaiabiodaZaqdcqGHris5aOWaaSaaaeaacqWGqbaucqWGnbqtdaWgaaWcbaGaemyAaKgabeaaaOqaaiabdwgaLjabdohaZjabdsha0jabdMgaPjabd2gaTjabdggaHjabdsha0jabdwgaLjabc6caUiabdcfaqjabd2eannaaBaaaleaacqWGPbqAaeqaaOGaeiOla4caaaaa@5338@

**Figure 1:**
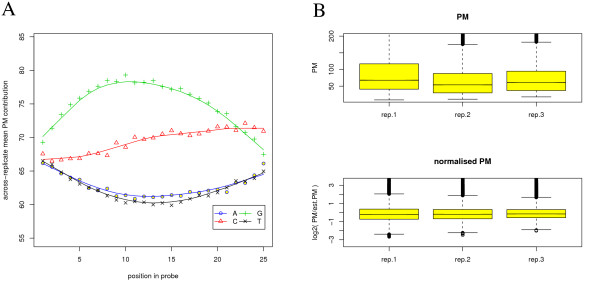
These plots summarise the Naef & Magnasco (2003) inspired normalisation procedure tested in the development of ExpressHMM. Sub-figure **A **displays fitted fluorescence contributions of the nucleotides A, C, G and T at each sequence position in the probe. Sub-figure **B **displays the effects of normalising chips using *PM .estimate*'s on the data distribution.

An illustration of this strategy and comparison with unnormalised distributions is shown in Figure 1. For the *PM *- *MM *approach rather than using an idealised *MM *we use the raw values of both *PM *and *MM*. A representative expression level over different replicates using the *MM *probe are computed as:

D¯:=13∑i=13(PMi−MMi).
 MathType@MTEF@5@5@+=feaafiart1ev1aaatCvAUfKttLearuWrP9MDH5MBPbIqV92AaeXatLxBI9gBaebbnrfifHhDYfgasaacH8akY=wiFfYdH8Gipec8Eeeu0xXdbba9frFj0=OqFfea0dXdd9vqai=hGuQ8kuc9pgc9s8qqaq=dirpe0xb9q8qiLsFr0=vr0=vr0dc8meaabaqaciaacaGaaeqabaqabeGadaaakeaadaqdaaqaaiabdseaebaacqGG6aGocqGH9aqpdaWcaaqaaiabigdaXaqaaiabiodaZaaadaaeWbqaaiabcIcaOiabdcfaqjabd2eannaaBaaaleaacqWGPbqAaeqaaOGaeyOeI0Iaemyta0Kaemyta00aaSbaaSqaaiabdMgaPbqabaGccqGGPaqkaSqaaiabdMgaPjabg2da9iabigdaXaqaaiabiodaZaqdcqGHris5aOGaeiOla4caaa@438C@

The performance of each normalisation strategy was evaluated (see discussion) and D¯
 MathType@MTEF@5@5@+=feaafiart1ev1aaatCvAUfKttLearuWrP9MDH5MBPbIqV92AaeXatLxBI9gBaebbnrfifHhDYfgasaacH8akY=wiFfYdH8Gipec8Eeeu0xXdbba9frFj0=OqFfea0dXdd9vqai=hGuQ8kuc9pgc9s8qqaq=dirpe0xb9q8qiLsFr0=vr0=vr0dc8meaabaqaciaacaGaaeqabaqabeGadaaakeaadaqdaaqaaiabdseaebaaaaa@2DCE@ was chosen as the more appropriate.

### Training data

**Figure 2:**
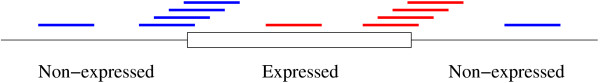
Labelling of probes. Each probe is labelled as either expressed (red) or non-expressed (blue) based on expression annotation of the genomic position corresponding to the 5' end of the probe.

From Ensembl (Ensembl Mart version 30) [22] we extract all annotated Human RefSeq transcripts [23] for the relevant ten chromosomes. Transcripts with no introns are discarded. To obtain a one-to-one correspondence between annotation and sequence, overlapping annotation of expression is collapsed, forming a single set representing all annotated expression. The training set consists of RefSeq genes each represented by the corresponding sequence of genomically consecutive probe scores. Using the 5' position of probes on the genome each probe is labelled as expressed or non-expressed in accordance with the collapsed RefSeq annotation (see Figure 2). The training set includes 6411 sequences of labelled probe scores that each represent the transcribed part of a RefSeq gene. The entire set is used for training the hidden Markov model (HMM). For evaluation purposes a ten-fold cross-validation is used.

### HMM Architecture and training

The correspondence between scores and annotated expression, presented by the training set, is captured by an HMM, see e.g. Durbin *et al. *[24]. An HMM is a probabilistic model that consists of a set of connected hidden states that each emit observables. In this case, states represent expression and non-expression and these states emit scores that constitute the sequence of probe scores over expressed or non-expressed regions. First order emission probabilities are used to model the dependency of scores for neighbouring-probes.

**Figure 3:**
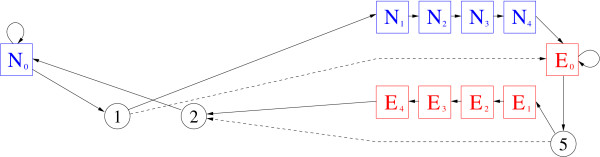
Core model. Boxes represent states emitting scores whereas circles represent linker states with no label and no emission. Arrows represent allowed transitions. States *E*_*i *_model expression and states *N*_*i *_model non-expression. The two colours used correspond to the two classes of states. Red states are trained on probes labelled as expressed. Blue states are trained on probes labelled as non-expressed.

**Figure 4:**
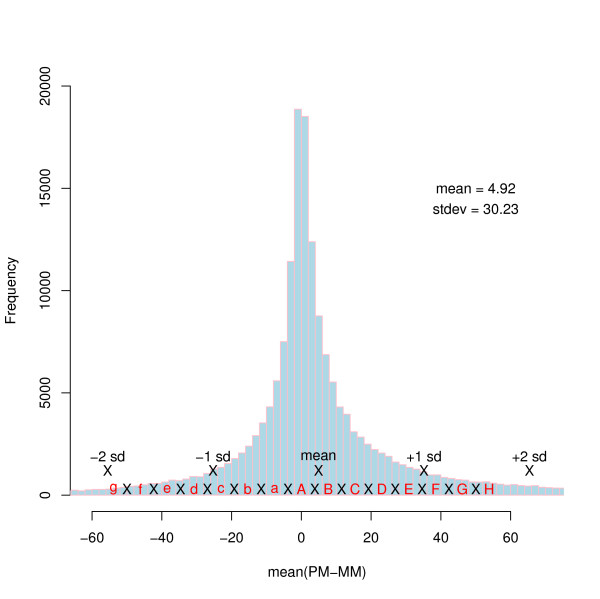
This figure shows how normalised expression scores are discretised prior to HMM training and decoding. 13 bins uniformly cover the scores from -50 to 50. Another two bins capture extreme positive and negative values.

The HMM is shown in Figure 3. Two loop states (*E*_0 _and *N*_0_) model the bulk of probe scores in expressed and non-expressed regions, capturing the score distributions of the two cases. The loop states are connected by states that capture score gradients induced by genomically consecutive probes that overlap borders of expressed regions. The loop probabilities are tied during training of the HMM and thus estimated as one parameter. This is done in order to avoid bias of predictions towards typical exon length. The HMM used is discrete and expression scores are binned. Considering the distributions of scores for probes annotated as expressed and non-expressed, we choose 13 bins uniformly covering the scores from -50 to 50. Another two bins capture extreme positive and negative values, (see Figure 4).

The type of HMM used is a Class HMM [25]. The collection of states in a Class HMM is divided into classes, each designated by a label. The observables (i.e. probe scores) in the training set are each assigned a label corresponding to a class. An observable with a given label can only be emitted by a state belonging to the class designated by that label. Hence, the labelling of states and observables in the training set determines which parts of the training data are used to estimate which parts of the model. In our case, the labels are expression and non-expression and correspond to the red and blue colours in Figure 3. The advantage of the Class HMM is that it allows all model parameters to be estimated simultaneously from sequences of labelled probe scores. Alternatively, the individual parts would have to be estimated separately from expressed and non-expressed probe scores. The model is trained using the Baum-Welch algorithm [26].

### Self-supervised re-training

**Figure 5:**
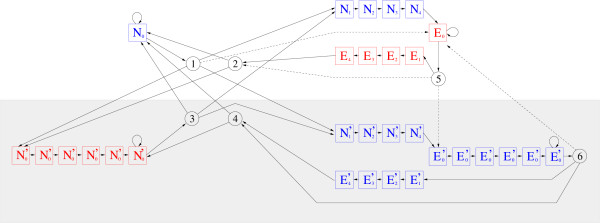
Core and shadow model. The upper part of the architecture is the core model shown and explained in Figure 3. The states E′i
 MathType@MTEF@5@5@+=feaafiart1ev1aaatCvAUfKttLearuWrP9MDH5MBPbIqV92AaeXatLxBI9gBaebbnrfifHhDYfgasaacH8akY=wiFfYdH8Gipec8Eeeu0xXdbba9frFj0=OqFfea0dXdd9vqai=hGuQ8kuc9pgc9s8qqaq=dirpe0xb9q8qiLsFr0=vr0=vr0dc8meaabaqaciaacaGaaeqabaqabeGadaaakeaacuWGfbqrgaqbamaaBaaaleaacqWGPbqAaeqaaaaa@2F52@ and N′i
 MathType@MTEF@5@5@+=feaafiart1ev1aaatCvAUfKttLearuWrP9MDH5MBPbIqV92AaeXatLxBI9gBaebbnrfifHhDYfgasaacH8akY=wiFfYdH8Gipec8Eeeu0xXdbba9frFj0=OqFfea0dXdd9vqai=hGuQ8kuc9pgc9s8qqaq=dirpe0xb9q8qiLsFr0=vr0=vr0dc8meaabaqaciaacaGaaeqabaqabeGadaaakeaacuWGobGtgaqbamaaBaaaleaacqWGPbqAaeqaaaaa@2F64@ in the shadow model, displayed in the shaded box, share emission parameters with the corresponding states *E*_*i *_and *N*_*i *_in the core model. This is done such that states in the shadow model do not contribute to estimation of the shared parameters. The labelling of states in the shadow model differs from those of the core model in that the states E′i
 MathType@MTEF@5@5@+=feaafiart1ev1aaatCvAUfKttLearuWrP9MDH5MBPbIqV92AaeXatLxBI9gBaebbnrfifHhDYfgasaacH8akY=wiFfYdH8Gipec8Eeeu0xXdbba9frFj0=OqFfea0dXdd9vqai=hGuQ8kuc9pgc9s8qqaq=dirpe0xb9q8qiLsFr0=vr0=vr0dc8meaabaqaciaacaGaaeqabaqabeGadaaakeaacuWGfbqrgaqbamaaBaaaleaacqWGPbqAaeqaaaaa@2F52@ are now labelled as non-expressed and N′0
 MathType@MTEF@5@5@+=feaafiart1ev1aaatCvAUfKttLearuWrP9MDH5MBPbIqV92AaeXatLxBI9gBaebbnrfifHhDYfgasaacH8akY=wiFfYdH8Gipec8Eeeu0xXdbba9frFj0=OqFfea0dXdd9vqai=hGuQ8kuc9pgc9s8qqaq=dirpe0xb9q8qiLsFr0=vr0=vr0dc8meaabaqaciaacaGaaeqabaqabeGadaaakeaacuWGobGtgaqbamaaBaaaleaacqaIWaamaeqaaaaa@2EF7@ is labelled as expressed. This allows the Baum-Welch algorithm to use the correspondence between annotation and score in proportion to how likely this is, given the information obtained in the initial training. E′0
 MathType@MTEF@5@5@+=feaafiart1ev1aaatCvAUfKttLearuWrP9MDH5MBPbIqV92AaeXatLxBI9gBaebbnrfifHhDYfgasaacH8akY=wiFfYdH8Gipec8Eeeu0xXdbba9frFj0=OqFfea0dXdd9vqai=hGuQ8kuc9pgc9s8qqaq=dirpe0xb9q8qiLsFr0=vr0=vr0dc8meaabaqaciaacaGaaeqabaqabeGadaaakeaacuWGfbqrgaqbamaaBaaaleaacqaIWaamaeqaaaaa@2EE5@ and N′0
 MathType@MTEF@5@5@+=feaafiart1ev1aaatCvAUfKttLearuWrP9MDH5MBPbIqV92AaeXatLxBI9gBaebbnrfifHhDYfgasaacH8akY=wiFfYdH8Gipec8Eeeu0xXdbba9frFj0=OqFfea0dXdd9vqai=hGuQ8kuc9pgc9s8qqaq=dirpe0xb9q8qiLsFr0=vr0=vr0dc8meaabaqaciaacaGaaeqabaqabeGadaaakeaacuWGobGtgaqbamaaBaaaleaacqaIWaamaeqaaaaa@2EF7@ states impose a minimum on the number of consecutive probe scores that can be captured by the shadow architecture. This means the model considers at least six consecutive probe scores at a time when evaluating the evidence in the training set. Prior to training the parameters are set so that the probability is distributed uniformly among transitions and among emissions for each state. The exceptions are the transitions shown as dashed lines. These are primed with probability 0.001.

As noted above, the correspondence between labelling of probe scores and actual expression in the relevant cell line is at best an approximation. This issue is addressed by re-training after adding a parallel shadow model that mirrors the core model shown in Figure 3. The combined model is shown in Figure 5. Each state in the shadow model, X′i
 MathType@MTEF@5@5@+=feaafiart1ev1aaatCvAUfKttLearuWrP9MDH5MBPbIqV92AaeXatLxBI9gBaebbnrfifHhDYfgasaacH8akY=wiFfYdH8Gipec8Eeeu0xXdbba9frFj0=OqFfea0dXdd9vqai=hGuQ8kuc9pgc9s8qqaq=dirpe0xb9q8qiLsFr0=vr0=vr0dc8meaabaqaciaacaGaaeqabaqabeGadaaakeaacuWGybawgaqbamaaBaaaleaacqWGPbqAaeqaaaaa@2F78@, shares emission probabilities with a corresponding state, *X*_*i*_, in the core model but does not contribute to the estimation of these parameters. The labelling of states in the shadow model differs from those of the core model in that the states E′i
 MathType@MTEF@5@5@+=feaafiart1ev1aaatCvAUfKttLearuWrP9MDH5MBPbIqV92AaeXatLxBI9gBaebbnrfifHhDYfgasaacH8akY=wiFfYdH8Gipec8Eeeu0xXdbba9frFj0=OqFfea0dXdd9vqai=hGuQ8kuc9pgc9s8qqaq=dirpe0xb9q8qiLsFr0=vr0=vr0dc8meaabaqaciaacaGaaeqabaqabeGadaaakeaacuWGfbqrgaqbamaaBaaaleaacqWGPbqAaeqaaaaa@2F52@ model non-expressed probes labelled as expressed and the N′0
 MathType@MTEF@5@5@+=feaafiart1ev1aaatCvAUfKttLearuWrP9MDH5MBPbIqV92AaeXatLxBI9gBaebbnrfifHhDYfgasaacH8akY=wiFfYdH8Gipec8Eeeu0xXdbba9frFj0=OqFfea0dXdd9vqai=hGuQ8kuc9pgc9s8qqaq=dirpe0xb9q8qiLsFr0=vr0=vr0dc8meaabaqaciaacaGaaeqabaqabeGadaaakeaacuWGobGtgaqbamaaBaaaleaacqaIWaamaeqaaaaa@2EF7@ state models expressed probes labelled as non-expressed. Additional states impose a minimum on the number of consecutive probe scores that can be captured by the shadow model. Without this constraint the model is not able to distinguish natural variation and noise from dubious annotation. The shadow component models regions annotated as expressed using the score distribution initially learned from non-expressed regions. Analogously regions annotated as non-expressed are modelled using the score distribution initially learned from expressed regions. Re-training after adding the shadow model allows the Baum-Welch algorithm to weight the contribution of training information to parameter estimation in proportion to how characteristic this is: In the E-step of the Baum-Welch algorithm the posterior probabilities of each emission from state *X*_*i *_are summed over all observables in the training set. The X′i
 MathType@MTEF@5@5@+=feaafiart1ev1aaatCvAUfKttLearuWrP9MDH5MBPbIqV92AaeXatLxBI9gBaebbnrfifHhDYfgasaacH8akY=wiFfYdH8Gipec8Eeeu0xXdbba9frFj0=OqFfea0dXdd9vqai=hGuQ8kuc9pgc9s8qqaq=dirpe0xb9q8qiLsFr0=vr0=vr0dc8meaabaqaciaacaGaaeqabaqabeGadaaakeaacuWGybawgaqbamaaBaaaleaacqWGPbqAaeqaaaaa@2F78@ state does not contribute to this sum. Hence, the posterior probability of using state *X*_*i *_relative to X′i
 MathType@MTEF@5@5@+=feaafiart1ev1aaatCvAUfKttLearuWrP9MDH5MBPbIqV92AaeXatLxBI9gBaebbnrfifHhDYfgasaacH8akY=wiFfYdH8Gipec8Eeeu0xXdbba9frFj0=OqFfea0dXdd9vqai=hGuQ8kuc9pgc9s8qqaq=dirpe0xb9q8qiLsFr0=vr0=vr0dc8meaabaqaciaacaGaaeqabaqabeGadaaakeaacuWGybawgaqbamaaBaaaleaacqWGPbqAaeqaaaaa@2F78@ for a given observable effectively constitutes a weight reflecting how likely the annotation is at the given position in the training set given the probe score distributions learned in the initial training. As a result, each subsequence of labelled probe scores in the training set contributes to estimation of emission probabilities to an extent determined by its reliability. It should be noted that the maximum likelihood of the model is obtained when only transition probabilities in the slave model are positive – the situation where the model discards all training material. The pre-training, however, produces a starting point that allows the model to reach a desirable local optimum.

Once the parameters of the HMM have been estimated the parallel shadow architecture is removed. The sequences of probe scores corresponding to the tiling of each chromosome are then decoded using an N-best algorithm [25]. In this step the most likely sequences of contiguous expressed and non-expressed probes are established. In addition, the forward-backward algorithm [27] is used to assign a posterior probability of expression to each probe. The prediction for each sequence of probe scores is then mapped onto the nucleotide sequence of the corresponding chromosome.

The tiling of the chromosomes only covers non-repeat sequence. Some predictions may span untiled regions. To avoid this, gaps in the tiling larger than ten bases are removed from the predictions.

### Evaluation of performance

To establish the relative performance of ExpressHMM and Affymetrix's normalisation and transfrag method (ANTM) we test each method on the training set of probes covering RefSeq genes. To ensure that test and training material do not overlap, the performance of ExpressHMM is evaluated using six-fold cross-validation. The ANTM predictions are mapped onto the training set for comparison.

To acknowledge the most recent repeat predictions we remove, from all genomic predictions, all overlaps to the Human Repeat Masker annotation from the UCSC genome browser database (October 2005) [28]. Performance statistics are calculated using the Eval program [29]. Sensitivity is defined as true positives divided by the sum of true positives and false negatives but is calculated as the number of annotation objects (e.g. nucleotides or sequence blocks) overlapped by predictions, divided by the total number of annotation objects. Specificity is defined as true positives divided by the sum of true positives and false positives but is calculated as the number of prediction objects overlapping annotation objects divided by the total number of prediction objects. The statistics are calculated this way to ensure a sensible treatment of one-to-many overlaps. Since the motivation in tiling array analysis is to find un-annotated expression a high specificity based on known expression is not a goal in itself. For this reason we report 1 – specificity as "Novel Prediction".

To further evaluate the performance we compare the two sets of predictions to annotation of Human ESTs, mRNAs, and known genes (UCSC Oct. 2005). Only annotation of tiled genomic sequence is included. Before evaluation the expression annotation is pooled and all overlapping annotation is collapsed into a maximal set where each nucleotide is only represented once.

Within both sets of annotation only a subset are actually expressed in the SK-N-AS cell line, and a substantial fraction of expression remains un-annotated. Collapsing all overlapping annotation presents a similar problem especially affecting splice site statistics. Relying on this annotation will make estimates of sensitivity artificially low and the absolute values of this statistic should not be given too much weight. The relative values for ExpressHMM and ANTM, however, should accurately reflect relative performances.

## Results

**Figure 6:**
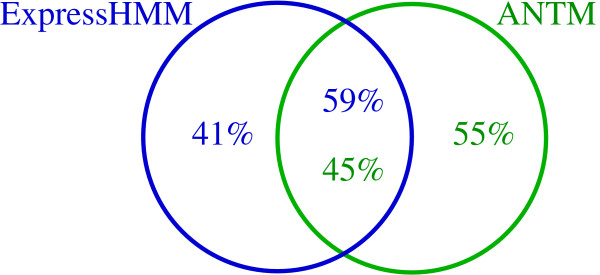
Overlap between ExpressHMM and ANTM transfrags predictions. The diagram shows the percentage of ExpressHMM predictions that are overlapped by one or more ANTM predictions and vice versa. Due to the one-to-many relationships of overlaps overlap percentages corresponding to each set of predictions are given.

**Figure 7:**
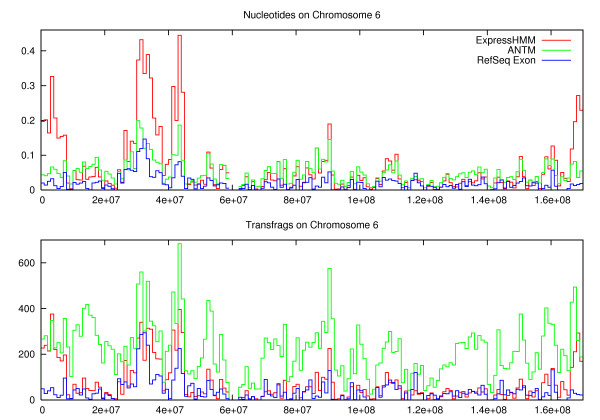
Distribution of predictions and annotation over chromosome 6. The top plot shows the fraction of nucleotides, within tiled portions of the genome, that are included in an ExpressHMM transfrag, ANTM transfrag, RefSeq exon, or the pool of EST, mRNA, and known genes. The bottom plot shows the number of ExpressHMM and ANTM transfrags as well as tiled parts of RefSeq exons. Bin size is one mega base.

The performance statistics obtained from the cross-validation of our model is shown in Table 1. Statistics of the ExpressHMM genomic predictions on the ten chromosomes are shown in Table 2. The overlap of ExpressHMM predictions to ANTM predictions is shown in Figure 6. The distribution of predictions and annotation is exemplified by chromosome 6 shown in Figure 7.

In addition to the most likely categorisation of probes ExpressHMM also calculates a posterior probability of expression for each probe. When mapped to the nucleotide sequence this results in an expression probability curve parallel to the predictions allowing the user to evaluate the significance of predictions and to directly view the evidence of expression.

**Figure 8:**
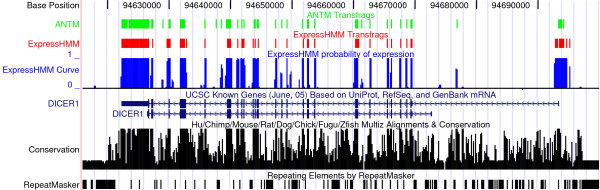
Predictions of the known *Dicer *gene involved in the processing of pre-miRNA to miRNA. The conservation curve shows the conservation in human, chimp, mouse, rat, dog, chicken, fugu, and zebrafish, based on a phylogenetic hidden Markov model [30]. Note how peaks in the ExpressHMM curve identify expression that is not sufficiently characteristic to result in a prediction. All information including the Repeat Masker track is extracted from the UCSC genome browser (October 2005).

**Figure 9:**
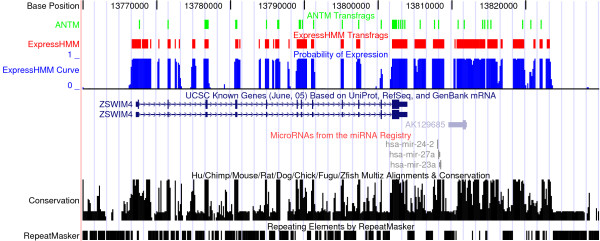
Predictions of the known *ZSWIM4 *gene and downstream expression including a miRNA cluster on the same strand. Note the correspondence between the conservation track and the ExpressHMM curve. The miRNA track is extracted from the UCSC genome browser (October 2005). It is generated by aligning miRNAs from the miRNA Registry [33] to the genome. The remaining tracks are explained in Figure 8.

Two example predictions, together with the ANTM predictions, are shown in Figure 8 and 9. Note the correspondence between ExpressHMM predictions and conservation in human, chimp, mouse, rat, dog, chicken, fugu, and zebrafish [30].

All predictions can be downloaded and viewed from our web site [2].

## Discussion

### Evaluation of normalisation strategies

In the process of developing our method we compared the performance of the *LL *and D¯
 MathType@MTEF@5@5@+=feaafiart1ev1aaatCvAUfKttLearuWrP9MDH5MBPbIqV92AaeXatLxBI9gBaebbnrfifHhDYfgasaacH8akY=wiFfYdH8Gipec8Eeeu0xXdbba9frFj0=OqFfea0dXdd9vqai=hGuQ8kuc9pgc9s8qqaq=dirpe0xb9q8qiLsFr0=vr0=vr0dc8meaabaqaciaacaGaaeqabaqabeGadaaakeaadaqdaaqaaiabdseaebaaaaa@2DCE@ normalisation strategies on the training set. Six-fold cross-validation was used to ensure no over-training. The nucleotide-level sensitivities of *LL *and D¯
 MathType@MTEF@5@5@+=feaafiart1ev1aaatCvAUfKttLearuWrP9MDH5MBPbIqV92AaeXatLxBI9gBaebbnrfifHhDYfgasaacH8akY=wiFfYdH8Gipec8Eeeu0xXdbba9frFj0=OqFfea0dXdd9vqai=hGuQ8kuc9pgc9s8qqaq=dirpe0xb9q8qiLsFr0=vr0=vr0dc8meaabaqaciaacaGaaeqabaqabeGadaaakeaadaqdaaqaaiabdseaebaaaaa@2DCE@ was approximately 38.8% and 44.6% respectively with corresponding specificities of 51.1% and 20.7%. Subsequent optimisation of the model resulted in further improvement of these values (see Table 2). The *LL *based normalisation is significantly more specific than the D¯
 MathType@MTEF@5@5@+=feaafiart1ev1aaatCvAUfKttLearuWrP9MDH5MBPbIqV92AaeXatLxBI9gBaebbnrfifHhDYfgasaacH8akY=wiFfYdH8Gipec8Eeeu0xXdbba9frFj0=OqFfea0dXdd9vqai=hGuQ8kuc9pgc9s8qqaq=dirpe0xb9q8qiLsFr0=vr0=vr0dc8meaabaqaciaacaGaaeqabaqabeGadaaakeaadaqdaaqaaiabdseaebaaaaa@2DCE@ approach but at a significant cost to sensitivity. We have used the D¯
 MathType@MTEF@5@5@+=feaafiart1ev1aaatCvAUfKttLearuWrP9MDH5MBPbIqV92AaeXatLxBI9gBaebbnrfifHhDYfgasaacH8akY=wiFfYdH8Gipec8Eeeu0xXdbba9frFj0=OqFfea0dXdd9vqai=hGuQ8kuc9pgc9s8qqaq=dirpe0xb9q8qiLsFr0=vr0=vr0dc8meaabaqaciaacaGaaeqabaqabeGadaaakeaadaqdaaqaaiabdseaebaaaaa@2DCE@ approach in this study because we view sensitivity as a more desirable trait than specificity for our predictor.

**Figure 10:**
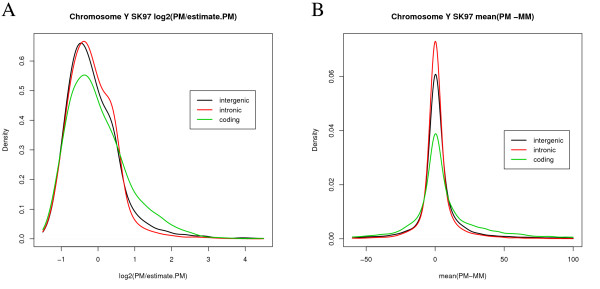
Sub-figure **A **shows the distribution of *LL *for exonic, intronic and intergenic regions from the Y chromosome. Sub-figure **B **shows the analogous distribution for D¯
 MathType@MTEF@5@5@+=feaafiart1ev1aaatCvAUfKttLearuWrP9MDH5MBPbIqV92AaeXatLxBI9gBaebbnrfifHhDYfgasaacH8akY=wiFfYdH8Gipec8Eeeu0xXdbba9frFj0=OqFfea0dXdd9vqai=hGuQ8kuc9pgc9s8qqaq=dirpe0xb9q8qiLsFr0=vr0=vr0dc8meaabaqaciaacaGaaeqabaqabeGadaaakeaadaqdaaqaaiabdseaebaaaaa@2DCE@. Note the heavy tails for both these statistics in the exonic distribution.

A tentative explanation for these rather surprising differences in performance can be found by considering Figure 10. Observe the heavy right tail of the *LL *distribution for probes tiling coding regions. For the D¯
 MathType@MTEF@5@5@+=feaafiart1ev1aaatCvAUfKttLearuWrP9MDH5MBPbIqV92AaeXatLxBI9gBaebbnrfifHhDYfgasaacH8akY=wiFfYdH8Gipec8Eeeu0xXdbba9frFj0=OqFfea0dXdd9vqai=hGuQ8kuc9pgc9s8qqaq=dirpe0xb9q8qiLsFr0=vr0=vr0dc8meaabaqaciaacaGaaeqabaqabeGadaaakeaadaqdaaqaaiabdseaebaaaaa@2DCE@ method both the left and right tails of the distribution are heavy for probes tiling coding regions. The HMM only requires significantly different distributions between expressed and non-expressed regions in order to perform well. The two heavy tails generated by the D¯
 MathType@MTEF@5@5@+=feaafiart1ev1aaatCvAUfKttLearuWrP9MDH5MBPbIqV92AaeXatLxBI9gBaebbnrfifHhDYfgasaacH8akY=wiFfYdH8Gipec8Eeeu0xXdbba9frFj0=OqFfea0dXdd9vqai=hGuQ8kuc9pgc9s8qqaq=dirpe0xb9q8qiLsFr0=vr0=vr0dc8meaabaqaciaacaGaaeqabaqabeGadaaakeaadaqdaaqaaiabdseaebaaaaa@2DCE@ method seems to supply more discriminative information to the HMM than the single heavy tail generated by the *LL *method. The effect of quantile normalisation was tested. This did not improve performance, and is not used for the results presented.

### Relative performances on the training set

As shown in Table 1, our probe sensitivity is higher than ANTM. Of the total number of probes annotated as expressed we predict 21% more than ANTM. Note that the upper limit to sensitivity is not 100% as only a fraction of the annotated sequence is actually expressed. For identification of exons ExpressHMM is equal to ANTM but better at predicting the borders of expressed regions correctly. The percentage of predicted probes that do not overlap RefSeq exons is 16% larger than ANTM. In contrast, the number of predicted transfrags not overlapping a RefSeq exon comprise only 27% of the ExpressHMM predictions whereas this number is 58% for ANTM. This means that even with the higher probe sensitivity ExpressHMM is more conservative than ANTM in predicting novel transfrags.

The HMM in ExpressHMM models the signal variability within expressed and non-expressed regions. As a result the algorithm does not predict very short expressed or non-expressed regions unless the relevant probe scores are highly characteristic. ANTM's threshold approach allows for little variability in score along a region. For these reasons ANTM tends to split exons into smaller predictions whilst ExpressHMM tends to join proximal exons into a single prediction. These differences are reflected in the length statistics given in Table 2. In effect, the two methods complement each other.

### Relative performances on the chromosome level

The comparison of genomic predictions to all Human EST, mRNA and known gene annotation within the tiled regions also reflects the good performance of ExpressHMM. The nucleotide sensitivity is 75% larger than ANTM's and our transfrag sensitivity is 23% larger. ExpressHMM identifies an 19% larger fraction of novel nucleotides. In contrast, however, the fraction of novel transfrags among ExpressHMM predictions is 24% smaller than among ANTM predictions.

ExpressHMM predicts roughly twice as many expressed nucleotides as ANTM. Still the total ExpressHMM predictions only amount to 1.5% of the genome. For comparison, recent cDNA based findings in Mouse indicates that 62% percent of the genome is transcribed and that the number of transcripts is at least an order of magnitude larger than the estimated number of genes [31].

As indicated by the nucleotide plot in Figure 7 the lower nucleotide specificity of ExpressHMM is not due to false positives uniformly distributed across the chromosomes. Rather, the distribution closely follows the density of both ANTM predictions and RefSeq exons. Note the excess of prediction in the sub-telomeric regions. This is most likely due to frequent duplication events in these regions as observed in the recent Chimpanzee-Human genome comparison [32]. These duplications are expected to cause extensive cross-hybridisation. The two large peaks around 4e+07 correspond to similar-sized peaks in the density of EST and mRNA annotation (data not shown).

The transfrag plot in Figure 7 shows the close correspondence between the distributions of ExpressHMM predictions and occurrence of RefSeq exons.

### Hidden Markov model

Individual values of probe scores form only a fraction of the signal characteristic of expressed and non-expressed regions. The distribution of scores within and between regions of expression constitutes an important source of information that is not utilised by simple threshold approaches. To capture as much relevant information as possible we use an adaptive approach. This learns directly from probe scores covering regions where expression is annotated. As a result the parameters of the model are optimal for the training data. These properties takes the place of the *ad hoc *cut-offs applied in previous approaches. 

Before settling on the HMM described above, a variety of models were tested. These included different order Markov models with and without modelling of transfrag borders. None of these offered apparent advantages over the one presented here. The order of the HMM has a pronounced effect on the results. For zeroth and first order emissions the nucleotide statistics are very similar. On the transfrag level, however, the zeroth order model is more sensitive (72% identified RefSeq exons) but less specific (55% novel transfrags predicted). We also experimented with excluding score sequences from the training set that did not seem unambiguously expressed. This improved the performance of some models but not that presented here.

The training of the model parameters does not include any nucleotide sequence information. In addition, to avoid any discriminative length modelling characteristic of coding exons the trained intron and exon length distributions are geometric and estimated as one to make them identical. As a result, the training data does not bias ExpressHMM predictions towards the length distribution of coding exons.

The expression probability curve that accompanies the ExpressHMM prediction has a direct interpretation as the evidence of expression given the model. It is a valuable tool to visually assess the significance of each transfrag as well as to identify regions with evidence not sufficient to result in a transfrag. With slight modifications ExpressHMM can equally well be used for the analysis of tiling micro arrays with non-overlapping probes of various length.

## Conclusion

In addition to performing better than the approach presented by Cheng *et al. *[1], the adaptive approach used by ExpressHMM avoids *ad hoc *thresholds for the analysis of signal data. Decisions regarding prediction are learned from the data in an automated fashion. In addition to predicting the most likely categorisation into expression and non-expression each genomic position queried by a probe is assigned a probability of expression. The resulting graph has a clearer interpretation than a smoothed fluorescence score. We expect that tiling arrays will play an increasing role in the investigation of genomic output.

## Authors' contributions

Kasper Munch and Paul P. Gardner contributed equally to the work presented, performing analyses, data acquisition, and preparing the manuscript. Peter Arctander and Anders Krogh contributed ideas and supervision.

## Figures and Tables

**Table 1:** Performance of ExpressHMM and ANTM on the training set of probes covering RefSeq genes. The statistics show to what extent the two approaches make predictions in accordance with the RefSeq annotation. The exon/transfrag statistics refer to the sets of contiguous probes representing exons/transfrags. The splice site sensitivities refer to the single probes constituting the borders of such sets.

	ExpressHMM	ANTM
Probe sensitivity	65%	44%
Novel probes predicted	65%	49%
Overlap exon Sensitivity	58%	58%
Novel transfrags predicted	27%	58%
5' Splice site sensitivity	16%	9%
3' Splice site sensitivity	16%	13%

**Table 2:** Performance of ExpressHMM and ANTM on the tiled regions of the ten chromosomes. The statistics show to what extent the two approaches make predictions in accordance with the collection of Human EST, mRNA, and known gene annotation.

	ExpressHMM	ANTM
Nucleotide sensitivity	35%	20%
Novel nucleotides	55%	46%
Nucleotide Count	43523440	21190998
Transfrag sensitivity	27%	22%
Novel transfrags	44%	58%
Transfrag Count	88604	170788
Average Length	491.21	124.08
Median Length	325	79
